# Intrinsic retinoic acid synthesis is required for oligodendrocyte progenitor expansion during CNS remyelination

**DOI:** 10.3389/fncel.2025.1550139

**Published:** 2025-02-24

**Authors:** Sonia E. Nanescu, Natacha M. Wathieu, Lauren Rosko, David S. Cha, Mahesh N. Kumar, Rafal T. Olszewski, Joan Reger, Maryna Baydyuk, Alisha N. Dua, Wojciech Krezel, Violetta Zujovic, Jeffrey K. Huang

**Affiliations:** ^1^Department of Biology, Georgetown University, Washington, DC, United States; ^2^Interdisciplinary Program in Neuroscience, Georgetown University, Washington, DC, United States; ^3^Institut de Génétique et de Biologie Moléculaire et Cellulaire, Illkirch-Graffenstaden, France; ^4^Sorbonne Université, Institut du Cerveau - Paris Brain Institute - ICM, Inserm, CNRS, APHP, Hôpital Pitié la Salpétrière University Hospital, DMU Neuroscience 6, Paris, France

**Keywords:** remyelination, oligodendrocyte progenitor cell (OPCs), microglia, multiple sclerosis, retinoic acid, Raldh2 (Aldh1a2)

## Abstract

Myelin regeneration (remyelination) in the CNS depends on the recruitment, proliferation and differentiation of oligodendrocyte precursor cells (OPCs) at demyelinated lesions. However, despite the presence of OPCs, very few oligodendrocytes and myelin are regenerated in chronic multiple sclerosis (MS) lesions for reasons that remain poorly understood. Here, using a spontaneous remyelination model in mice, we found that retinaldehyde dehydrogenase 2 (Raldh2), a rate-limiting enzyme for retinoic acid (RA) synthesis, is upregulated in OPCs and in a subpopulation of microglia/macrophages during remyelination. Tamoxifen induced deletion of Raldh2 globally, or conditionally in OPCs, resulted in significantly fewer proliferating OPCs in lesions, leading to decreased oligodendrocyte numbers and myelin density. Moreover, induced deletion of Raldh2 globally also resulted in increased microglia/macrophage density in lesions. Further, exogenous RA delivery into lesions significantly increased oligodendrocyte lineage cells, while also decreasing proinflammatory microglia/macrophages, with no significant effect on anti-inflammatory microglia/macrophages. Postmortem MS brain sections revealed Raldh2 was absent in the majority of OPCs in chronic inactive lesions compared to the other lesion types. These results suggest that Raldh2 upregulation in lesions is critical for OPC proliferation during remyelination, and reveal that the failure to regenerate sufficient oligodendrocytes and myelin in chronic MS lesions may arise from impaired OPC expansion due to the failure to intrinsically synthesize RA.

## Introduction

Multiple sclerosis (MS) is an immune-driven neurodegenerative disease characterized by chronic inflammation, demyelination and axonal injury in the central nervous system (CNS) (Compston and Coles, 2008; Trapp and Nave, 2008; Reich et al., 2018). Myelin regeneration (remyelination) becomes increasingly difficult at the later stage of the disease, in which chronic CNS inflammation coupled with regenerative failure results in irreparable axonal dystrophy, and widespread neurodegeneration (Franklin, 2002; Lassmann et al., 2012; Dutta and Trapp, 2014). Exactly why regenerative failure occurs in MS remains unclear (Wolswijk, 2000; Franklin, 2002; Hagemeier et al., 2012; Boyd et al., 2013). One possibility is that oligodendrocyte precursor cells (OPCs), once migrated into lesions, fail to expand, leading to insufficient mature oligodendrocytes in lesions for remyelination (Wolswijk, 1998, 2000). However, the mechanisms driving OPC proliferation within the injury microenvironment remains poorly understood.

Retinoic acid (RA) is a highly conserved signaling molecule that stimulates stem/progenitor cell proliferation and differentiation during the development and regeneration of certain tissues and organs (Chambon, 1996; Ogura et al., 1996; Maden and Hind, 2003; Maden, 2007; Duester, 2013). RA promotes signaling by binding to retinoic acid receptors (RARα, RARβ and RARγ) and their heterodimeric partners retinoid X receptors (RXRα, RXRβ and RXRγ) to activate or suppress the transcription of a suite of target genes by binding to promoters containing the retinoic acid response element (RARE) (Rossant et al., 1991; Chambon, 1996; Kumar and Duester, 2011). RXRs, particularly RXRγ, are highly expressed in oligodendrocyte lineage cells and macrophages during remyelination in rodent CNS lesions, and in MS plaques (Huang et al., 2011). The loss of RXRγ expression in mice prevents efficient oligodendrocyte differentiation and remyelination, suggesting that RA signaling plays a critical role in CNS remyelination. However, for RA signaling to occur, RA must be available in demyelinated lesions to drive oligodendrocyte lineage cell progression. During development, RA is widely distributed in the embryonic CNS, and regulates tissue patterning and corticogenesis (Siegenthaler et al., 2009; Duester, 2013). In the adult CNS, RA level is significantly reduced and produced primarily by the meninges, and this is thought to influence homeostatic function through a paracrine mechanism (Zhang et al., 2003; Siegenthaler et al., 2009). However, the cellular source of RA involved in remyelination remains unknown. Since RA signaling is upregulated in oligodendrocyte lineage cells during remyelination, we hypothesize that RA synthesis occurs locally within lesions to stimulate RA signaling in oligodendrocyte lineage cells during remyelination.

RA is generated from retinaldehyde through retinaldehyde dehydrogenase activity, following a series of oxidation reactions from vitamin A. The mammalian CNS expresses at least three types of retinaldehyde dehydrogenase—Raldh1, Raldh2 and Raldh3 (Li et al., 2000; Wagner et al., 2002). Raldh2 is predominantly expressed in the central nervous system (CNS), with its presence in meningeal cells playing a crucial role in brain development (Zhang et al., 2003; Siegenthaler et al., 2009; Haushalter et al., 2017). Additionally, our recent findings indicate that Raldh2 expression in the meninges supports the production and differentiation of oligodendrocyte precursor cells (OPCs) in the corpus callosum (Morrison et al., 2020). Following spinal cord injury, Raldh2 has also been detected in NG2^+^ cells, and suggested to promote a permissive environment for axon outgrowth (Mey et al., 2005; Goncalves et al., 2018). However, the precise identity of these NG2^+^ cells in injury remains unclear, and whether they are involved in remyelination remains unknown. Here, we examine the role of Raldh2 in remyelination following focal demyelinating injury in mice. We found that Raldh2 is upregulated in oligodendrocyte lineage cells and a subpopulation of microglia/macrophages in remyelinating lesions and required for OPC proliferation and subsequent oligodendrocyte differentiation after demyelination. Moreover, administration of RA into demyelinated lesions stimulated OPC proliferation, leading to increased mature oligodendrocytes in lesions. We also found that Raldh2 was absent in the majority of OPCs in chronic inactive lesions in postmortem MS brain sections. Our results suggest that intrinsic RA synthesis in adult OPCs is necessary to drive oligodendrocyte lineage cell expansion in demyelinated lesions, and that the loss of RA synthesis in OPCs may result in the failure to regenerate sufficient oligodendrocytes and myelin in chronic MS lesions.

## Materials and methods

### Mice

C57BL/6 and *CMV-CreER*^*T*2^, *NG2-Cre* and *Pdgfra-cre/ERT2* mice were purchased from The Jackson Laboratory (Bar Harbor, ME). *Raldh2* floxed mice were obtained from IGBMC, Strasbourg, France. *Raldh2^loxP/loxP^*;*CMVCre-ERT* were bred by crossing Raldh2 floxed mice with *B6.129-Gt(ROSA)26Sortm1(cre/ERT2)Tyj/J* (Jackson Laboratory). To induce conditional deletion of Raldh2, 1 mg of 4-hydroxytamoxifen (4OHT) was administered daily by i.p., injections for 4 consecutive days. Animals were allowed to recover for 4 more days before inducing the demyelination lesion. The retinoic acid reporter mouse line Tg(RARE-Hspa1b/lacZ)12Jrt/, which expresses beta-galactosidase (lacZ) gene under the control of the retinoic acid responsive element (RARE), was purchased from Jackson Laboratory (Bar Harbor, ME, USA). Both males and females were used for experiments, with genders in the experimental groups distributed as equally as possible. Experiments were performed according to approved Institutional Animal Care and Use Committee (IACUC) protocols of Georgetown University.

### Chemicals

4OHT was purchased from Sigma. 500 μl of 2 mg/ml emulsion were injected daily using peanut oil as vehicle. RA (all trans) was obtained from Sigma and dissolved in dimethyl sulfoxide, then stored at −80°C in the dark. All subsequent dilutions were also kept shielded from light.

### Lysolecithin-induced spinal cord demyelination

Injection of lysolecithin into the spinal cord ventral funiculus was performed between T10 and T11 thoracic vertebrae to create focal demyelination in mice (Jeffery and Blakemore, 1995; Baydyuk et al., 2019). To this end, incision of back skin and muscle was followed by removal of muscles and tendons between adjacent thoracic vertebrae with forceps to expose the spinal cord. The dura was then punctured with a fine needle before injection. A pulled glass needle glued to the tip of a 10 ul Hamilton syringe attached to a micromanipulator was used to deliver 0.5 μl 1.0% lysolecithin (Sigma) into the ventral funiculus of the mouse spinal cord to induce a focal demyelinated lesion. After injection, the muscles overlying the lesion were sutured, which will later help identify the site of lesions, followed by interrupted sutures of the overlying skin with a non-absorbable suture (Ethilon 5.0). For pain management, the analgesic Carprofen was delivered subcutaneously for 3 consecutive days. Extended release buprenorphine was also delivered. The mice were monitored for at least 72 h after surgery for signs of distress and pain before perfusion with 4.0% paraformaldehyde at 7, 14 and 21 days postlesion.

### Immunohistochemistry

After 4.0% paraformaldehyde (PFA) perfusion, spinal cords were dissected and post fixed for 30 min at room temperature, followed by overnight immersion in 20% sucrose in PBS before embedding in OCT compound and frozen in dry ice. 12 μm sections were generated on Superfrost plus glass slides, and focal demyelination was identified by toluidine blue staining before tissue collection and then storage at −80° until immunostaining. Prior to staining, tissues were allowed to dry for 30 min and incubated for 1 h in the blocking buffer containing 5% normal goat serum and 0.3% Triton^Tm^ X-100 made in PBS. Primary antibodies: rabbit Raldh2 (1:200, Abcam) rat anti-CD11b (1:100; AbD Serotec), mouse anti-iNOS/NOS Type II (1:100; BD Pharmingen), chicken Arg1 (1:1000, Millipore), mouse Nkx2.2 (1:100, DSHB) mouse APC/CC1 (1:100, Millipore), mouse and rabbit Olig2 (1:100 and 1:300, respectively, Millipore) and mouse Olig2 (1:100, Millipore), rat MBP4 (1:400, AbD Serotec), rabbit Raldh2 (1:200, Abcam), rabbit Ki67 (1:1000, Thermo Fisher), rat PDGFRα (1:400, BD Pharminogen), rabbit NF 200 (1:100, Sigma), Smi32 (1;1000, Calbiochem), GFAP (1:500, Sigma), rabbit anti-cleaved caspase-3 (1:100; Cell Signaling Technology) were incubated overnight at 4% Celsius in same blocking buffer. Antigen retrieval was used for Olig2, Raldh2, and mouse on mouse antigen retrieval with M.O.M.™ kit (Vector Laboratories) for Nkx2.2 and CC1. For double labeling of Raldh2 and Cd11b, iNOS, or Arginase1, tissue was permeabilized by immersion at −20°C for 10 min in a 1:1 mixture of Acetone and Methanol. Next day, slides were washed 3 times with TBS-tween solution (0.05% Tween) for 10 min, and the following secondary antibodies added for 1 h: Alexa Fluor^®^ 488 IgG (1:500), Alexa Fluor^®^ 594 Goat Anti-Mouse IgG (1:500), Texas Red Goat Anti-Mouse IgG (1:500). After 3 more washes in TBS-tween of 10 min each, slides were mounted with Fluoromount-G (SouthernBiotech). For quantification of immunohistochemical staining, cells were manually counted from low magnification, non-overlapping images. The lesioned area was defined by hyper nuclear (DAPI) staining. Corrected fluorescence for SMI-32 and NF200 was determined by measuring the integrated intensity, subtracting the product of the selected region’s area and the mean background fluorescence as described previously (Psachoulia et al., 2016). A minimum of three sections from *n* = 3–5 mice were analyzed. The proportion or density of cells was determined per mouse, and the average and standard error were calculated for each group.

### *In situ* hybridization

Tissues were perfused and collected in the same manner as described above. The primers used for cloning the Raldh2 *in situ* probe had the sequence AGC TCC TGC CGT CGC CCA CG (forward) and CA CTC CAA TGG GCT CGT GTC (reverse) according to a sequence published previously (Zhao et al., 1996). The protocol used was according to the *in situ* hybridization procedure previously described (Pringle et al., 1992; Huang et al., 2011).

### Beta-galactosidase staining

Retinoic acid reporter mice were perfused with 0.2% glutaraldehyde in PBS and spinal cords postfixed for 10 min on ice, followed by 3 consecutive washes in LacZ wash solution (2 mM MgCl_2_, 0.01Na Deoxycholate and 0.02% Nonidet P-40 in PBS). Whole mount spinal cords were then incubated for two hours in LacZ wash with 5 mg/ml X-Gal, 5 mM ferrocyanide and 5 mM ferricyanide added to the wash solution to allow enzymatic reaction at 37°C in the dark. Spinal cords were then immersed in sucrose, frozen and sectioned.

### Postmortem MS tissue sections

Human brain tissue was provided by the UK multiple sclerosis tissue bank (Richard Reynolds, Imperial College, London). Donor informed consent was obtained via a prospective donor scheme following ethical approval by the London Multicenter research ethics committee (MREC 02/2/39). Snap frozen sections of MS patient postmortem tissue was processed at the cryostat with 12 μm sections and rehydrated with PBS prior to any staining. Luxol fast blue immunostaining was performed in conjunction with MHC class II peroxidase conjugated staining to identify and classify the different types of lesions. In order to perform immunohistochemistry with anti-Kp1 (1:100, DAKO), anti-Sox10 (1:100, R&D Systems) and anti-Raldh2 (1:50, Abcam) antibodies, brain sections were microwaved in Low pH unmasking solution (Vector Laboratories) then pre-incubated 1 h with a blocking solution buffer (10% normal goat serum, 0.1% Triton in PBS) at room temperature before the incubation overnight with the primary antibodies. After overnight incubation, slides were extensively washed in 0.1% Triton in PBS and incubated with appropriate secondary antibodies.

### Statistics

Data were analyzed for statistical significance with GraphPad Prism 6 by one-way ANOVA followed by Bonferroni post-hoc for multiple comparisons. For each comparison, averages from 2 to 5 lesions from a group of 3–6 mice were analyzed. Statistical significance is reported as *P ≤ 0.05, **P ≤ 0.01, ***P ≤ 0.001, ****P < 0.0001.

## Results

### Upregulation of Raldh2 in oligodendrocyte lineage cells and a subpopulation of microglia/macrophages during remyelination

**FIGURE 1:**
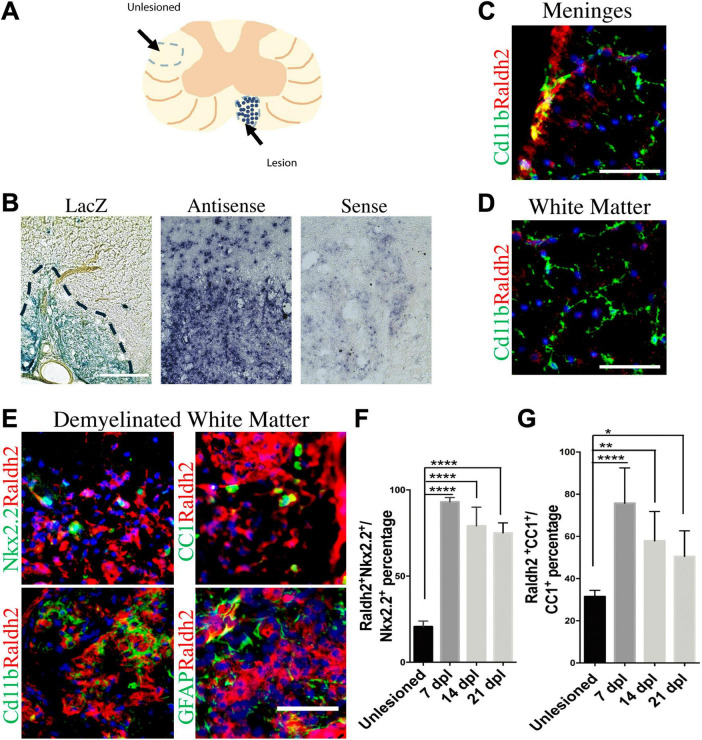
Induction of Raldh2 expression during remyelination. **(A)** Diagram displaying lesioned and unlesioned white matter analyzed. **(B)** β-gal staining in lesioned spinal cord of RARE-LacZ reporter mouse at 14 dpl. Lesioned outlined in black. Scale bar 30 μm. **(C)** *In situ* hybridization of mouse lesioned spinal cord with antisense or sense probe against *Raldh2* at 14 dpl. Scale bar, 50 μm. **(D)** Representative images for Raldh2 (red) and Cd11b labeled resting microglia (green) at meninges and non-lesioned white matter. Scale bar, 50 μm. **(E)** Immunodetection of Raldh2 co-labeling with Nkx2.2 for OPCs, CC1 for oligodendrocytes, Cd11b for activated microglia/macrophages, and GFAP for reactive astrocytes at 14 dpl. Scale bar, 100 μm. **(F)** Percentage of Raldh2 labeled OPCs and **(G)** oligodendrocytes in unlesioned and lesioned tissue at 7, 14, 21 dpl. One-way ANOVA with Tukey post-hoc for multiple comparisons. Data presented as mean + s.d. ****P < 0.0001, **P < 0.01, * *P* < 0.05.

To track RA signaling during remyelination, lysolecithin-induced spinal cord demyelination (Figure 1A) was performed on the *RARE-LacZ* transgenic RA reporter mouse line (Rossant et al., 1991), in which the beta-galactosidase (LacZ) gene is driven under control of the retinoic acid response element (RARE) promoter, followed by enzymatic detection of LacZ expression at 14 days postlesion (dpl). We found that beta-galactosidase staining was primarily observed within demyelinated lesions and rarely detected in non-lesioned white matter (Figure 1B). This result suggested that the activation of RA signaling is restricted to lesions after CNS injury and raised the question whether RA synthesis is induced locally in response to injury to stimulate RA signaling. Since retinaldehyde dehydrogenases are critical for RA synthesis, and retinaldehyde dehydrogenase 2 (Raldh2) has previously been shown to regulate CNS function in development and injury (Aoto et al., 2008; Siegenthaler et al., 2009; Goncalves et al., 2018), we next determined if Raldh2 has a role in CNS remyelination. To examine Raldh2 expression, *in situ* hybridization was performed on lesioned spinal cord sections at 7 dpl. We found that the antisense RNA against *Raldh2* was strongly detected in demyelinated lesions compared to sense control (Figure 1C), suggesting that RA synthesis was induced locally in demyelinated lesions.

**FIGURE 2:**
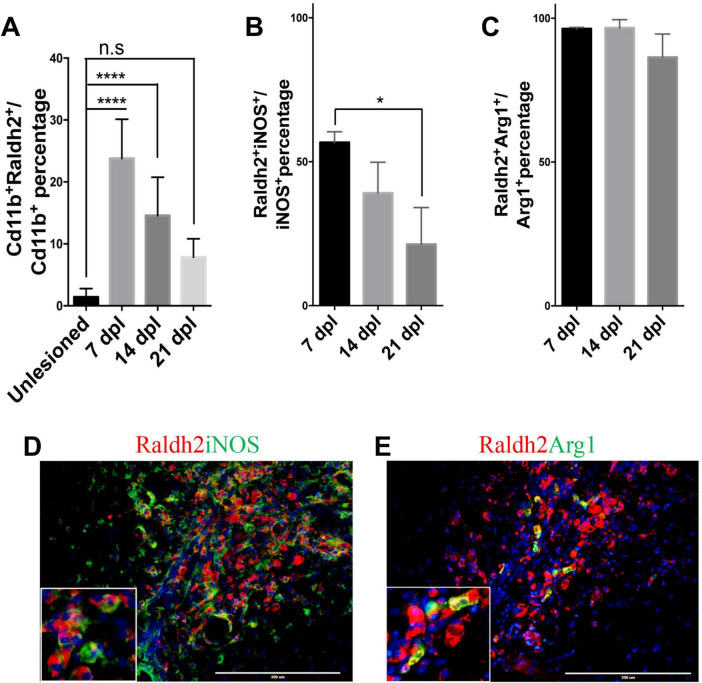
Raldh2 expression in microglia subtypes during remyelination. **(A)** Percentage of Raldh2^+^ microglia/macrophages in unlesioned white matter, and at 7, 14, and 21 dpl. Percentage of **(B)** Raldh2 labeled iNOS^+^ microglia/macrophages and **(C)** Raldh2 labeled Arg1^+^ microglia/macrophages in lesions at 7, 14, and 21 dpl. Representative image of **(D)** Raldh2^+^iNOS^+^ and **(E)** Raldh2^+^Arg1^+^ microglia/macrophages at 7 dpl. Scale bar, 200 μm. One way ANOVA with Tukey post-hoc for multiple comparisons. Data presented as mean + s.d. ****P < 0.0001, *P < 0.05, n.s., not significant.

To determine which cell populations in lesions expressed Raldh2 during remyelination, co-immunostaining analysis of demyelinated spinal cord tissues for Raldh2 and Nkx2.2^+^ OPCs, CC1^+^ oligodendrocytes, Cd11b^+^ microglia/macrophages, or GFAP^+^ astrocytes was performed at 7, 14 and 21 dpl, corresponding to OPC recruitment/proliferation, oligodendrocyte differentiation, and remyelination, respectively (Huang et al., 2011). In non-lesioned tissue, we found Raldh2 was most strongly expressed in the meninges and weakly expressed in the white matter (Figure 1D). However, at the lesion site, strong Raldh2 expression was detected in Nkx2.2^+^ OPCs, CC1^+^ oligodendrocytes, and Cd11b^+^ microglia/macrophages, but not GFAP^+^ reactive astrocytes (Figure 1E). Quantification revealed Raldh2 was expressed in the majority of OPCs and oligodendrocytes in lesions compared to non-lesioned white matter in all three postlesion timepoints analyzed (Figures 1F, G). Moreover, Raldh2 was detected in approximately 25% of total Cd11b^+^ microglia/macrophages in lesions at 7 dpl, but the percentage was reduced at subsequent timepoints (Figure 2A). To determine if Raldh2 expression in microglia/macrophage corresponded with their activation states, co-immunostaining analysis for Raldh2 and induced nitric oxide (iNOS) corresponding to pro-inflammatory microglia/macrophages, or arginase 1 (Arg1) corresponding to anti-inflammatory microglia/macrophages (Miron et al., 2013; Psachoulia et al., 2016) was performed. We found that Raldh2 was detected in about 50% of pro-inflammatory macrophages at 7 dpl, and that its expression in these cells was significantly reduced by 21 dpl (Figures 2B, D). Moreover, we found that Raldh2 was detected in nearly 100% of anti-inflammatory macrophages across all three postlesioned timepoints (Figures 2C, E). These results demonstrate that Raldh2 is induced in lesions following demyelination and primarily expressed in oligodendrocyte lineage cells during remyelination.

### *Raldh2* loss-of-function in adult tissues impairs oligodendrocyte lineage cell expansion and promotes CNS inflammation

**FIGURE 3:**
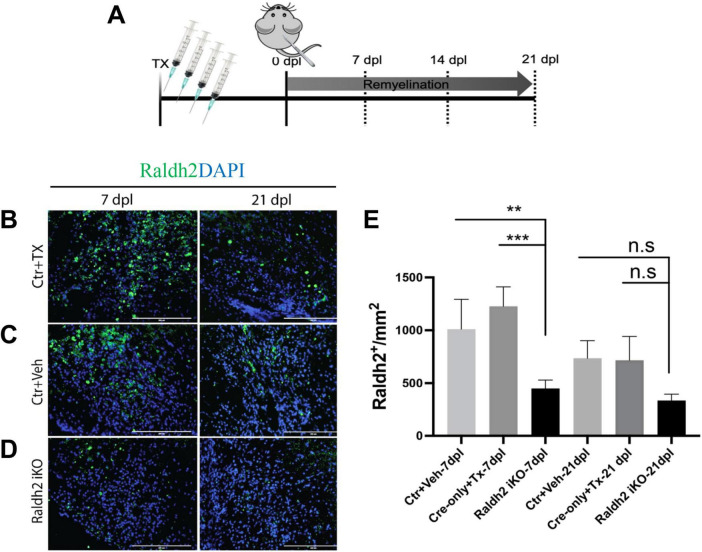
Assessment of 4-hydroxy tamoxifen (4OHT) efficiency in demyelinated lesion of *Raldh2-iKO* mouse. **(A)** Diagrams illustrating the inducible *Raldh2* deletion strategy. 4OHT was injected for four consecutive days into *CMV-Cre-ER*^*T*2^;*Raldh2^loxP/loxP^* (*Raldh2-iKO*), and *Cre-ER*^*T*2^ (Ctr+Tx) control mice. Additionally, peanut oil instead of 4OHT was injected into *Cre-ER*^*T*2^;*Raldh2^loxP/loxP^* mice as a second control (Ctr+Veh). Immunostaining for Raldh2 expression in lesions of **(B)** Ctr+TX, **(C)** Ctr+Veh, and **(D)** Raldh2 iKO at 7 and 21 dpl. **(E)** Quantification of Raldh2^+^ cells in lesions per mm^2^ shows statistically significant reduction of Raldh2 expressed cells at 7 dpl. Scale bar, 200 μm. ANOVA with Tukey post-hoc for multiple comparisons. Data presented as mean + s.d. ***P < 0.001, **P < 0.01.

We next determined if Raldh2 expression is required for oligodendrocyte lineage cell progression following demyelinating injury. Since *Raldh2* knockout in mice is embryonically lethal (Niederreither et al., 1999; Mic et al., 2002) and therefore precludes the analysis of its function in the adult CNS, we generated an inducible *CMV-Cre-ER*^*T*2^;*Raldh2^loxP/loxP^* mouse line (*Raldh2-iKO*) that allowed for tamoxifen-induced deletion of the l*oxP*-flanked *Raldh2* in adult tissues prior to demyelination. The *Raldh2-iKO* mice expressed at least one copy of the *CMV-cre-ER*^*T*2^ allele and both copies of the *loxP* site flanked *Raldh2* allele. To induce *Raldh2* deletion in adult tissues, intraperitoneal (i.p.) injections of 4-hydroxy-tamoxifen (4OHT) into mice was performed for four consecutive days, followed by four days of rest to allow sufficient time for 4OHT to trigger *Raldh2* excision before lysolecithin-induced spinal cord demyelination (Figure 3A). As controls, *CMV-Cre-ER*^*T*2^;*Raldh2^loxP/loxP^* mice injected with peanut oil (Ctr+Veh), and *CMV-Cre-ER*^*T*2^ injected with 4OHT (Ctr+Tx) were lesioned. Following demyelination, all mice were sacrificed at either 7, 14, or 21 dpl for immunohistochemistry analysis. To assess the efficiency of 4OHT induced deletion, immunostaining analysis of Raldh2 expression was performed on demyelinated lesions. We found that the *Raldh2-iKO* mice exhibited a 48–67% reduction of Raldh2 expression in the lesioned area at 7 dpl compared to both controls (Figures 3B–E), suggesting that 4OHT induced the loss of *Raldh2* expression in about half of the adult cell population in demyelinated lesions.

**FIGURE 4:**
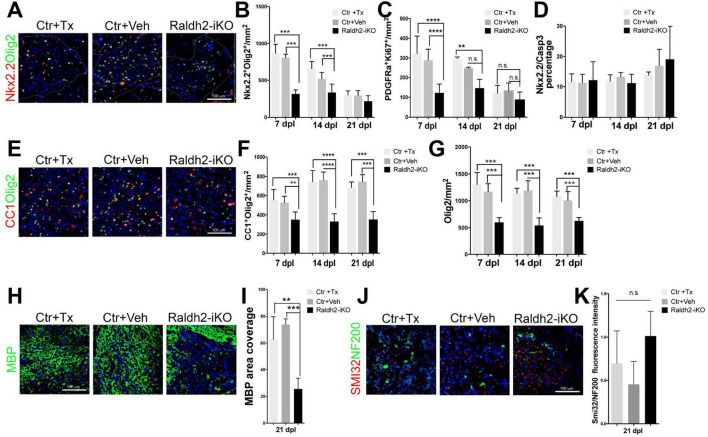
Induced deletion of *Raldh2* globally in adult mice reduces oligodendrogenesis after demyelination. Lesions of Ctr+TX and Ctr+Veh controls, and *Raldh2-iKO* mice were analyzed at 7, 14, 21 dpl. **(A)** Representative images of Nkx2.2^+^Olig2^+^ OPCs at 14 dpl. Quantification of **(B)** Nkx2.2^+^Olig2^+^ OPCs, **(C)** PDGFRα^+^Ki67^+^ proliferating OPCs, and **(D)** cleaved Caspase-3^+^Nkx2.2^+^ apoptotic OPCs in lesions at 7, 14, 21 dpl. **(E)** Representative images of CC1^+^Olig2^+^ oligodendrocytes at 14 dpl. Quantification of **(F)** CC1^+^Olig2^+^ oligodendrocytes, and **(G)** Olig2^+^ oligodendrocyte lineage cells in lesions at 7, 14, 21 dpl. **(H)** Representative images of myelin basic protein (MBP) staining at 21 dpl, corresponding to remyelination, and **(I)** quantification of MBP area coverage in lesions at 21 dpl. **(J)** Representative images of SMI32^+^NF200^+^ labeling at 14 dpl, corresponding to dystrophic axons, and **(K)** quantification of SMI32 staining normalized to NF200 in lesions at 14 dpl. Scale bar, 100 μm. One-way ANOVA with Tukey post-hoc for multiple comparisons except for unpaired two-tailed Student’s *t*-test in **(I)**. Data presented as mean + s.d. ****P < 0.0001, ***P < 0.001, **P < 0.01, n.s., not significant.

To examine the impact of *Raldh2* loss-of-function on oligodendrocyte lineage cells in demyelinated lesions, co-immunostaining analysis was performed. We found that the *Raldh2-iKO* mice displayed a significant reduction in the number of Nkx2.2^+^Olig2^+^ OPCs in lesions at 7 and 14 dpl compared to the control groups (Figures 4A, B). At 21 dpl, the relative number of OPCs in lesions was low in all three mouse groups compared to the previous timepoints, and not significantly different from each other. This observation was expected, as most OPCs would normally have differentiated into mature oligodendrocytes by 21 dpl. To determine if impaired OPC proliferation contributed to the reduction of OPCs in lesions of the *Raldh2-iKO* mice, co-immunostaining analysis for Ki67 and PDGFRα (another OPC marker) was performed. We found that the relative number of Ki67^+^ OPCs in lesions was significantly reduced in the *Raldh2-iKO* group by greater than 50% at 7 dpl compared to the control groups (Figure 4C). Furthermore, their density remained relatively constant across all three postlesion timepoints, suggesting the OPCs that migrated to the lesion were unable to proliferate. Moreover, we did not detect a significant difference in the number of Caspase3^+^Nkx2.2^+^ OPCs in lesions between the *Raldh2-iKO* group and the control groups in any of the postlesion timepoints analyzed (Figure 4D), suggesting that *Raldh2* loss-of-function did not affect apoptosis. These results indicated that *Raldh2* expression is necessary for OPC proliferation during remyelination. Next, to determine if the failure of OPCs to proliferate in lesions affected subsequent oligodendrocyte differentiation or remyelination, CC1^+^Olig2^+^ co-immunolabeling of oligodendrocytes was performed. We found that the relative density of oligodendrocytes was significantly reduced in the *Raldh2-iKO* compared to controls (Figures 4E, F). Moreover, the density of oligodendrocytes in lesions remained constant across all three timepoints, suggesting that impaired OPC proliferation resulted in fewer mature oligodendrocytes in lesions. Indeed, quantification of Olig2 immunolabeling in lesions revealed a significant reduction of total oligodendrocyte lineage cells in *Raldh2-iKO* mice compared to controls in all three postlesion timepoints (Figure 4G). This reduction also resulted in decreased MBP immunostaining in lesions (Figures 4H, I), suggesting that OPC proliferation through RA signaling is necessary for the expansion of oligodendrocyte lineage cell numbers in lesions during remyelination. Although the relative number of oligodendrocyte lineage cells in lesions was reduced in *Raldh2-iKO* mice, we did not observe an increase in axonal dystrophy in lesions at 21 dpl, as shown by the relative distribution of SMI32^+^NF200^+^ axons in lesions (Figures 4J, K).

**FIGURE 5:**
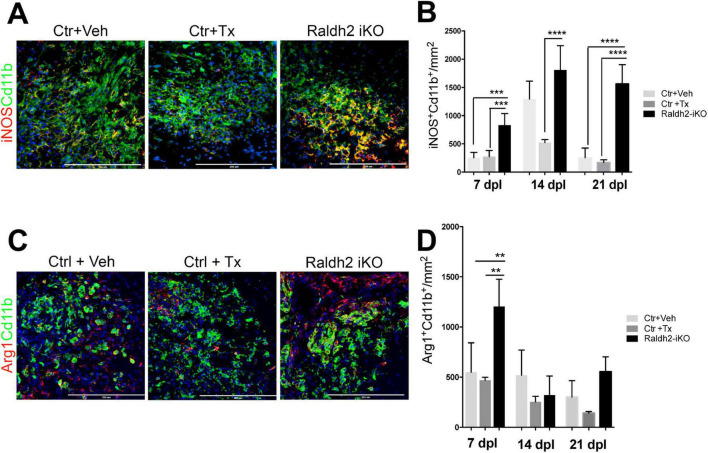
Induced deletion of Raldh2 globally in adult mice increases inflammation after demyelination. Lesions of Ctr+TX and Ctr+Veh controls, and *Raldh2-iKO* mice were analyzed at 7, 14, 21 dpl. **(A)** Representative images of iNOS^+^Cd11b^+^ microglia/macrophages at 14 dpl, and **(B)** quantification of iNOS^+^Cd11b^+^ microglia/macrophages in lesions at 7, 14, 21 dpl. **(C)** Representative images of iNOS^+^Arg1^+^ microglia/macrophages at 14 dpl, and **(D)** quantification of Arg1^+^Cd11b^+^ microglia/macrophages in lesions at 7, 14, 21 dpl. Scale bar, 200 μm. One-way ANOVA with Tukey post-hoc for multiple comparisons. Data presented as mean + s.d. ****P < 0.0001, ***P < 0.001, **P < 0.01, n.s., not significant.

Since Raldh2 is also expressed in a subpopulation of microglia/macrophages during remyelination, we next investigated the effect of Raldh2 loss-of-function on microglia/macrophage activation after demyelination. We found that following demyelination, the *Raldh2-iKO* group displayed increased density of iNOS^+^Cd11b^+^ macrophages in lesions compared to controls. Quantification showed that the number of iNOS^+^Cd11b^+^ macrophages in lesions of KO mice was 2-fold greater at 7 dpl, and 10-fold greater at 21 dpl than controls, suggesting that inflammation remained elevated in lesions in the absence of Raldh2 expression (Figures 5A, B). Moreover, we found that the relative density of anti-inflammatory macrophages, which display Arg1^+^Cd11b^+^ expression, was initially greater in the *Raldh2-iKO* group compared to controls at 7 dpl, but their density in lesions was reduced to similar levels as controls at 14 and 21 dpl (Figures 5C, D). These results suggest that RA synthesis in microglia/macrophages may be necessary to regulate their activation state during remyelination.

### Conditional deletion of *Raldh2* in OPCs reduces OPC proliferation and the expansion of oligodendrocyte lineage cells

**FIGURE 6:**
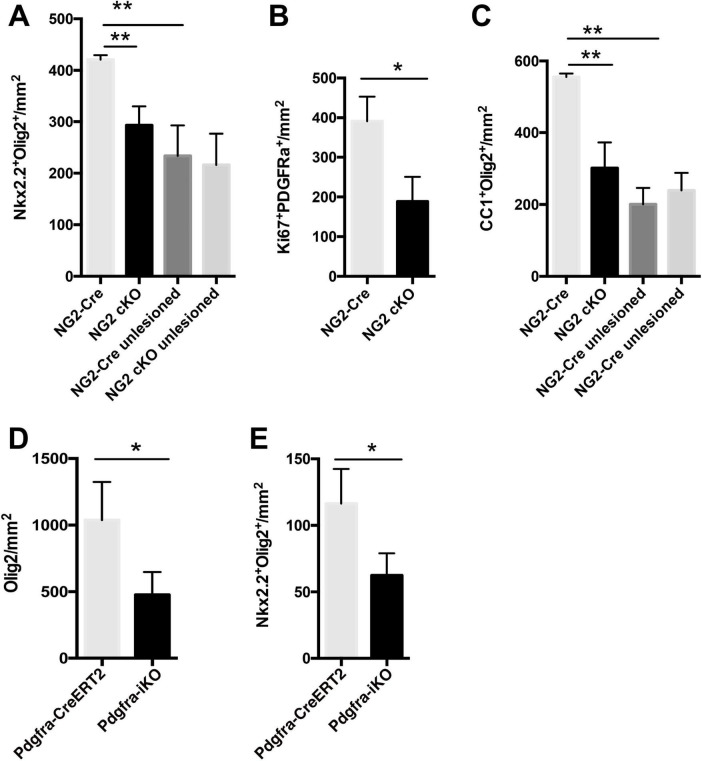
Conditional deletion of Raldh2 in OPCs reduces oligodendrogenesis after demyelination. Quantification of **(A)** Nkx2.2^+^Olig2^+^ OPCs, **(B)** PDGFRα^+^Ki67^+^ proliferating OPCs, and **(C)** CC1^+^Olig2^+^ oligodendrocytes in lesioned and adjacent unlesioned white matter of *NG2-Cre;Raldh2^loxP/loxP^* (NG2-cKO) and NG2-Cre (control) mice at 14 dpl after focal demyelination. Quantification of **(D)** Olig2^+^ oligodendrocyte lineage cells, and **(E)** Nkx2.2^+^Olig2^+^ OPCs in lesions in *Pdgfra-CreERT2;Raldh2^fl/fl^* (*Pdgfra-iKO*), and *Pdgfra-CreERT2* (control) mice at 14 dpl following 4OHT injection and focal demyelination. One-way ANOVA with Tukey post-hoc for multiple comparisons except for unpaired two-tailed Student’s *t*-test in **(B,D,E)**. Data presented as mean + s.d. **P < 0.01, *P < 0.05, n.s., not significant.

Since 4OHT induced deletion of Raldh2 in *Raldh2-iKO* likely occurred in both oligodendrocyte lineage cells and microglia/macrophages, it was not possible to determine if the observed decrease of oligodendrocyte numbers in lesions resulted from Raldh2 loss-of-function in OPCs, or microglia/macrophages. To address this issue, Raldh2 floxed mice were crossed with *NG2-Cre* mice to generate a *NG2-Cre;Raldh2^loxP/loxP^* mouse line (*NG2-cKO*), in which *Raldh2* was conditionally deleted in OPCs during development. The *NG2-cKO* mice grew to adulthood and did not appear to display obvious gross abnormalities. To determine if conditional deletion of *Raldh2* in OPCs affects oligodendrocyte lineage cell progression in demyelinated lesions, lysolecithin-induced demyelination was performed on *NG2-cKO*, and *NG2-Cre* mice (control), followed by immunostaining analysis of lesions at 14 dpl. We found that the *NG2-cKO* group exhibited a significant reduction in number of Nkx2.2^+^Olig2^+^ OPCs, by approximately 25%, in demyelinated lesions compared to control (Figure 6A). Moreover, we found that the relative density of OPCs in unlesioned white matter tissue was not significantly different between the *NG2-cKO* group and control, suggesting that the conditional deletion of *Raldh2* in OPCs only affected OPCs that have migrated to the lesion. To determine if OPC proliferation is dependent on Raldh2 expression in OPCs, co-immunostaining analysis for Ki67 and PDGFRα expression was performed. We found that the number of proliferating OPCs was significantly reduced, by approximately 50%, in the lesions of the *NG2-cKO* group compared to control (Figure 6B). Moreover, the number of CC1^+^Olig2^+^ labeled oligodendrocytes was also reduced by approximately 50%, in lesions (Figure 6C). These results suggest the deletion of *Raldh2* in NG2^+^ cells decreased OPC proliferation, leading to reduced mature oligodendrocytes in demyelinated lesions.

However, since NG2 may be transiently expressed by a subpopulation of activated macrophages under excitotoxic CNS lesions (Bu et al., 2001), it remained possible that NG2-Cre mediated excision of Raldh2 during development might affect macrophage activity in lesions. To confirm that Raldh2 expression in OPCs and not NG2^+^ macrophages is required for OPC proliferation, *Raldh2^loxP/loxP^* mice were crossed with *Pdgfra-creERT2* mice to generate a *Pdgfra-cre/ERT2*;*Raldh2^loxP/loxP^* (*Pdgfra-iKO*) mouse line, which allowed the conditional deletion of *Raldh2* in adult OPCs by 4OHT administration prior to demyelinating injury. Cre-mediated excision of *Raldh2* is not expected to occur in macrophages because they do not express PDGFRα (Bu et al., 2001). As control, 4OHT was injected into *Pdgfra-CreERT2* mice. We found that, after 4OHT administration and lysolecithin-induced demyelination, the relative numbers of Olig2^+^ oligodendrocyte lineage cells (Figure 6D), as well as Nkx2^+^Olig2^+^ OPCs (Figure 6E) in lesions were significantly reduced, by approximately 50%, in *Pdgfra-iKO* mice compared to control. These results, together with our results from the analysis of NG2-cKO mice confirmed that *Raldh2* expression in adult OPCs is required for OPC proliferation during remyelination. Moreover, our results suggest that RA is synthesized in OPCs intrinsically in response to injury and activates RA signaling.

### Administration of RA promotes oligodendrocyte lineage cell expansion and inhibits pro-inflammatory microglia/macrophage activation

**FIGURE 7:**
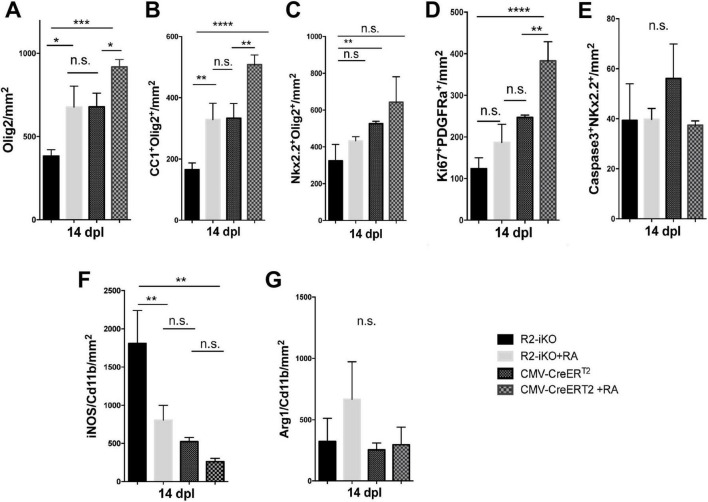
RA injection into demyelinated lesions increases oligodendrogenesis. Quantifications of **(A)** Olig2^+^ oligodendrocyte lineage cells, **(B)** CC1^+^Olig2^+^ oligodendrocytes, **(C)** Nkx2.2^+^Olig2^+^ OPCs, **(D)** Ki67^+^PDGFRα^+^ proliferating OPCs, **(E)** Caspase3^+^Nkx2.2^+^ apoptotic OPCs, **(F)** iNOS^+^Cd11b^+^ microglia/macrophages, and **(G)** Arg1^+^Cd11b^+^ microglia/macrophages in lesions of *Raldh2-iKO* (R2-iKO) and *CMV-CreERT2* mice with or without RA injection at 14 dpl. One-way ANOVA with Tukey post-hoc for multiple comparisons. Data presented as mean+s.d. ****P < 0.0001, ***P < 0.001, **P < 0.01, *P < 0.05, n.s., not significant.

Since RA is a diffusible ligand that activates RA receptor signaling, we next asked if exogenously applied RA could promote oligodendrocyte lineage cell progression in demyelinated lesions of mice lacking *Raldh2* expression. To test this, we locally injected RA into demyelinated lesions of 4OHT-treated *Raldh2-iKO* mice and compared them to those without RA injection as controls. We found that a single dose of RA (10^–5^ M) significantly increased the number of Olig2^+^ oligodendrocyte lineage cells in lesions of *Raldh2-iKO* compared to un-injected mice (Figure 7A). Additionally, RA injection led to a two-fold increase in Nkx2.2^+^Olig2^+^ OPCs and CC1^+^Olig2^+^ oligodendrocytes in lesions (Figures 7B, C). We also found that OPC proliferation, as indicated by co-localized Ki67 and PDGFRα expression, appeared to be increased following RA injection. However, no statistical significance was observed between RA injected *Raldh2-iKO* and control lesions at 14 dpl (Figure 7D). This result may be expected, as OPC proliferation in lesions typically peaks at earlier postlesion timepoints, and Ki67 expression may already be reduced by 14 dpl. Further, the relative number of apoptotic OPCs in lesions at 14 dpl, based on Caspase3^+^Nkx2.2^+^ co-labeling, did not differ significantly between RA-injected and un-injected *Raldh2-iKO* mice (Figure 7E), indicating that RA injection did not influence OPC survival. These results suggest that exogenous RA rescues the deficit of oligodendrocyte lineage cell numbers in *Raldh2*-deficient mice.

We next examined whether RA injection could also enhance oligodendrocyte cell numbers in mice with normal *Raldh2* expression. To this end, RA was injected locally into the spinal cord of *CMV-CreER*^*T*2^ mice, which retain both copies of *Raldh2*, and analyzed the effects at 14 dpl. We found that the number of Olig2^+^ oligodendrocyte lineage cells in lesions increased by more than 25% compared to *CMV-CreER*^*T*2^ mice without RA injection (Figure 7A). Moreover, RA injection significantly increased both Nkx2.2^+^Olig2^+^ OPCs and CC1^+^Olig2^+^ oligodendrocytes in lesions (Figures 7B, C). Analysis of Ki67 and PDGFRα co-labeling in lesions revealed that RA injection significantly increased the number of proliferating OPCs, even at 14 dpl (Figure 7D). However, there was no statistically significant difference in apoptotic OPCs between RA-injected and un-injected mice (Figure 7E), suggesting that exogenous RA did not enhance OPC survival. Together, these results suggest that increasing RA concentration in lesions has an additive effect on oligodendrocyte lineage cell numbers. Further, a single dose of RA in demyelinated lesions was sufficient to promote the expansion of the entire oligodendrocyte lineage cell population.

Since *Raldh2* loss-of-function in adult CNS also led to increased pro-inflammatory microglia/macrophages in lesions, we next determined if RA injection into demyelinated lesions influences microglia/macrophage activation in *Raldh2-iKO* mice. We found that the co-injection of RA with lysolecithin into 4OHT treated *Raldh2-iKO* mice significantly reduced the number of iNOS^+^Cd11b^+^ microglia/macrophages in lesions compared to those without RA treatment (Figure 7F). Additionally, RA injection did not significantly affect the number of Arg1^+^Cd11b^+^ microglia/macrophages in lesions (Figure 7G). These results suggest that exogenous RA administration suppresses pro-inflammatory macrophage activation in Raldh2-deficient mice. To further assess whether increased RA concentrations influence macrophage activation, we injected RA into lesions of control *CMV-CreERT2* mice. However, we found that elevated RA levels did not significantly impact the relative densities of pro-inflammatory or anti-inflammatory microglia/macrophages in lesions (Figures 7F, G). These findings suggest that while microglia/macrophages in lesions remained unresponsive to increased RA, elevated RA levels primarily promoted OPC proliferation, thereby accelerating myelin repair.

### Reduced Raldh2 expressed OPCs in chronic inactive multiple sclerosis lesions

**FIGURE 8:**
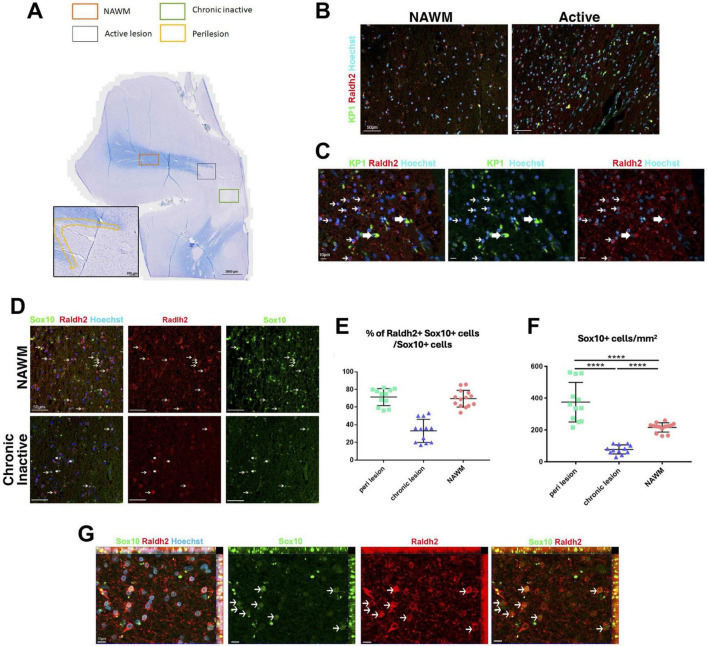
Raldh2 expression in MS patient brain sections. **(A)** Luxol fast blue staining combined with major histocompatibility complex II peroxidase immunolabeling showing chronic inactive lesion (green square), an active lesion (gray square), perilesion area (yellow highlight) and NAWM (orange square) where quantifications were performed. **(B)** Representative images of human samples for KP1^+^Raldh2^+^ microglia/macrophages in normal appearing white matter (NAWM) and active lesion. Scale bar, 50 μm. **(C)** Higher magnification of active lesions, in which thin arrows indicate Raldh2^+^Kp1^–^ cells, and thick arrows indicate Raldh2^+^KP1^+^ cells. Scale bar, 10 μm. **(D)** Representative images of Raldh2^+^Sox10^+^ OPCs in NAWM and chronic inactive lesions. Thin arrows indicate Raldh2^+^Sox10^+^ cells, and thick arrows indicate Raldh2^+^Sox10^–^ cells. Scale bar, 50 μm. **(E)** Percentages of Raldh2^+^Sox10^+^ OPCs among total Sox10^+^ cells in perilesions, chronic lesion, and NAWM. **(F)** Quantification of Sox10^+^ OPCs per mm^2^ within perilesions, chronic lesions, and NAWM. **(G)** Representative image of Raldh2^+^Sox10^+^ OPCs in perilesions. Arrows point to Raldh2^+^Sox10^+^ OPCs. Scale bar, 10 μm. One-way ANOVA and Bonferroni post-hoc test. Data presented as mean ± s.e.m. ****P < 0.0001.

The presence of reduced but viable OPCs, and decreased oligodendrocytes in the lesions of *Raldh2* deficient mice resembled the pathology of chronic MS lesions, which often display variable number of OPCs, and a severe deficiency in oligodendrocytes and remyelinated axons (Chang et al., 2000; Wolswijk, 2000). Although the cause of this pathology remains unclear, the failure to regenerate oligodendrocytes in lesions has been postulated to contribute to remyelination failure in MS (Franklin, 2002; Franklin and Ffrench-Constant, 2008). This prompted us to determine if Raldh2 expression is altered in the brain of patients with multiple sclerosis (MS). To this end, we characterized the expression of Raldh2 in microglia/macrophages and OPCs in active lesions (site of ongoing demyelination and early-stage repair), perilesions (site of later stage repair), chronic inactive lesions (site of failed repair), and normal appearing white matter (NAWM) from postmortem MS brain sections (Figure 8A). We found that Raldh2 appeared to be expressed in all KP1^+^ macrophages in all the lesion types examined (Figures 8B, C), suggesting that RA synthesis in microglia/macrophages was not impaired in MS. Moreover, we detected Raldh2 expression in approximately 70% of Sox10^+^ OPCs in NAWM and perilesion (Figures 8D, E). However, Raldh2 was detected in only approximately 35% of Sox10^+^ OPCs in chronic lesions, suggesting that a significant portion of OPCs in chronic lesions no longer synthesized RA. Furthermore, the total numbers of Sox10^+^ OPCs in chronic lesions were significantly smaller than those in NAWM and perilesions (Figures 8D, F), suggesting that the expansion of oligodendrocyte lineage cells was not achieved to the same extent as in normal tissue or active lesions. Interestingly, we also found a greater number of Sox10^+^ OPCs in perilesions compared to NAWM (Figure 8F). These OPCs displayed stronger Raldh2 staining intensity, and more branched morphology compared to those in the other lesion types (Figure 8G), suggesting that OPCs in perilesions might be more activated, and undergoing proliferation or differentiation. Together, these results reveal the deficiency of oligodendrocytes and remyelinated axons in chronic MS lesions may arise from failed OPC expansion, due to the loss of intrinsic Raldh2 expression.

## Discussion

We found that Raldh2 is significantly upregulated in OPCs and in a subpopulation of microglia/macrophages during remyelination. Our results suggest that local RA synthesis within lesions is crucial for driving OPC proliferation for oligodendrocyte regeneration and remyelination. Previous *in vitro* studies have shown that exogenous RA application to OPC or spinal cord cultures regulates oligodendrocyte lineage cell function (Barres et al., 1994; Noll and Miller, 1994), but its role *in vivo* remained unclear. Moreover, it was unknown if RA regulates oligodendrocyte lineage cells via a paracrine or intracrine mechanism. Our observation that Raldh2 expression in OPCs stimulates their proliferation within demyelinated lesions indicates that RA signaling in OPCs is achieved through an intracrine mechanism under a CNS injury environment. This differs from the homeostatic role of RA on the CNS, in which neural cell function is regulated through a paracrine mechanism (Aoto et al., 2008; Chen and Napoli, 2008). Since Raldh2 is expressed in oligodendrocyte lineage cells and a subset of microglia/macrophages in demyelinated lesions, this intracrine mechanism may serve as an injury response essential for promoting RA signaling within OPCs and microglia/macrophages in the lesions, while avoiding effects on cells outside the lesion. Further, given RA is a lipid soluble metabolite, additional mechanisms may be in place to prevent the RA synthesized in OPCs or microglia/macrophages from diffusing out of the cells and into non-injured tissues. It may be possible that cellular retinoic acid binding proteins or RA catabolizing enzymes are present in the OPC and microglia/macrophage cytoplasm to ensure that RA is sequestered or degraded in the cytoplasm and only active inside the nucleus, thus limiting the diffusion of RA out of cells.

The mechanisms driving Raldh2 expression in OPCs and microglia/macrophages following injury remain unclear. Several growth factors, including PDGF-A, FGF2 and LIF, are known to regulate OPC proliferation during remyelination (Hinks and Franklin, 1999; Armstrong, 2007; Deverman and Patterson, 2012). Moreover, recent studies have shown that factors released from microglia/macrophages or regulatory T-cells can influence oligodendrocyte lineage cell progression during remyelination (Miron et al., 2013; Psachoulia et al., 2016; Dombrowski et al., 2017; Baydyuk et al., 2020). Given the abundance of inflammatory cells in CNS lesions during the early stages of remyelination, inflammatory cytokines may induce Raldh2 expression within demyelinated lesions. For example, TNF-alpha has been shown to stimulate OPC proliferation during remyelination, indicating a dual role in promoting both inflammation and OPC proliferation (Arnett et al., 2001). Additionally, IL-4, which is associated with anti-inflammatory activity, has been shown to facilitate oligodendrocyte differentiation and remyelination (Psachoulia et al., 2016; Zhang et al., 2019). IL-4 is known to work synergistically with RA in macrophages to stimulate Raldh2 expression through a feedforward mechanism (Broadhurst et al., 2012; Lee et al., 2016). Whether a similar mechanism occurs in oligodendrocyte lineage cells during remyelination is still unclear.

How Raldh2 regulates oligodendrocyte remyelination remains to be determined. During vertebrate development, RA is known to interact with Sonic Hedgehog (Shh) to coordinate cell proliferation and patterning (Ogura et al., 1996; Ribes et al., 2006). In adults, Shh maintains neural stem cells and regulates oligodendrocyte development during postnatal myelination (Ahn and Joyner, 2005; Tong et al., 2015). Our recent findings indicate that Raldh2 plays a role in Shh signaling during OPC development in the mouse corpus callosum (Morrison et al., 2020). Since Shh signaling in oligodendrocyte lineage cells is critical for remyelination (Ferent et al., 2013; Samanta et al., 2015; Tong et al., 2015; Sanchez et al., 2018; Russo et al., 2024), potentially mediated through Shh co-receptors like Boc and modulation of Wnt pathways (Zakaria et al., 2019; Ming et al., 2020), it is plausible that Raldh2 influences remyelination via the Shh pathway. In support of this, a recent study demonstrated that Shh activation, through genetic activation of Smoothened (Smo) promotes OPC proliferation (Nocera et al., 2024). Additionally, Megalin (LRP2), a multifunctional endocytic receptor, is known to play a critical role in RA metabolism by influencing Shh signaling (Liu et al., 1998; Ortega et al., 2012; Brożyna et al., 2022). It has been shown that Megalin regulates Shh-induced OPC proliferation and migration during development, and this may involve transient expression of Megalin in astrocytes that facilitates Shh presentation to OPCs or regulating its gradient during development (Ortega et al., 2012). Collectively, these findings suggest the crosstalk between RA, Shh, and Megalin may be critical in regulating oligodendrocyte myelination and repair.

Additionally, we found that most Arg1+ microglia/macrophages in lesions also expressed Raldh2 during remyelination. It is well established that iNOS+ microglia/macrophages, associated with a pro-inflammatory state, decrease, while Arg1+ microglia/macrophages, linked to an anti-inflammatory state, increase during remyelination (Miron et al., 2013; Psachoulia et al., 2016; Hu et al., 2024). The role of Raldh2 in anti-inflammatory microglia/macrophages remains unclear. RA has been shown to modulate macrophage activity by suppressing inflammatory mediators, including the release of TNF-α and nitric oxide (NO) (Mathew and Sharma, 2000; Oliveira et al., 2018). Moreover, RA has been demonstrated to synergize with IL4 to activate anti-inflammatory pathways and facilitate the phenotypic transition of macrophages from a pro-inflammatory to an anti-inflammatory state (Vellozo et al., 2017; Pinos et al., 2023). The presence of Raldh2 in anti-inflammatory microglia/macrophages suggest intrinsic RA synthesis may contribute to this transition during remyelination. Further studies are needed to explore how inflammatory cytokines regulate RA synthesis and signaling in OPCs and microglia/macrophages within demyelinated lesions, which may provide deeper insight into the mechanisms of myelin repair.

Our results indicate that RA signaling is crucial for both OPC proliferation and inflammation resolution. Injection of RA into demyelinated lesions significantly increased OPC proliferation and reduced pro-inflammatory macrophage density, leading to enhanced oligodendrocyte differentiation and remyelination. Interestingly, while RA application promotes OPC proliferation, 9-cis-retinoic acid (9cRA), an RA isomer, does not affect OPCs but instead enhances oligodendrocyte differentiation (Huang et al., 2011). This suggests different RA receptors may be necessary at distinct stages of oligodendrocyte lineage cell progression. Since RA binds to RARs, and 9cRA has a preference for RXRs, it is possible that OPC proliferation in lesions is dependent on RAR signaling (Laeng et al., 1994; Kim et al., 2017), whereas oligodendrocyte differentiation is dependent on RXR signaling. Indeed, 9cRA mediated RXRγ signaling has been demonstrated to promote oligodendrocyte differentiation during remyelination (Huang et al., 2011), and this effect has been shown to be mediated through the heterodimerization of RXRγ with vitamin D receptor (VDR) (de la Fuente et al., 2015).

Interestingly, we detected Raldh2 expression in OPCs and microglia/macrophages within normal-appearing white matter (NAWM) of MS patients, in contrast to its expression pattern in the mouse CNS, where Raldh2 is mostly absent in non-lesioned tissues except in the meninges (Morrison et al., 2020). This discrepancy may be attributed to the smaller size of the mouse brain, where meningeal-derived RA can sufficiently diffuse into the parenchyma and regulate CNS homeostasis via paracrine signaling. In contrast, the larger human brain may require intrinsic RA synthesis by parenchymal cells to maintain homeostasis. However, in response to injury, local upregulation of RA within lesions may be necessary to expand oligodendrocyte lineage cells to facilitate CNS repair in a timely manner. Notably, our observation that Raldh2 expression is absent in most OPCs within chronic MS lesions, compared to its presence in active lesions and NAWM, suggests that the chronic inactive lesion environment may affect Raldh2 expression in OPCs. A failure to expand OPC populations within lesions likely results in fewer mature oligodendrocytes and insufficient remyelination, contributing to progressive neurodegeneration and disease severity. Indeed, MS patients exhibit reduced plasma retinol levels and altered RA receptor expression, linking RA availability to clinical disease activity (Royal et al., 2002).

In summary, we found that the loss of Raldh2 expression in OPCs significantly impairs their proliferation following demyelinating injury, and this defect leads to a reduction in mature oligodendrocytes in lesions. These results highlight the critical role of RA signaling in myelin repair. Therapeutic strategies targeting OPC expansion or preventing Raldh2 loss may improve remyelination in MS and slow disease progression.

We report that the induction of retinoic acid (RA) synthesis through Raldh2 in adult oligodendrocyte precursor cells (OPCs) occurs locally at the injury site and is necessary to stimulate the expansion of OPCs and mature oligodendrocytes during remyelination. Moreover, Raldh2 expression is absent in the majority of OPCs in chronic inactive lesions of multiple sclerosis (MS) patients. This work suggests that the deficiency of mature oligodendrocytes and myelinated axons in chronic MS lesions might arise from the failure to expand OPCs in lesions, and that therapeutic approaches to promote OPC proliferation by increasing RA levels in chronic demyelinated lesions might overcome myelin regenerative failure in MS.

## Data Availability

The raw data supporting the conclusions of this article will be made available by the authors, without undue reservation.
